# Green Fabrication of Keratin Nanoparticles from Yak Horn by Steam Flash Explosion: Structure–Property Evolution

**DOI:** 10.3390/biom16071044

**Published:** 2026-07-17

**Authors:** Zhisong Qian, Haiyue Feng, Kun Meng, Xiaoyong Chen, Zhen Hong Chang, Gege Yan, Jiayu Cheng, Mohd Shaiful Sajab, Peer Mohamed Abdul, Gongtao Ding, Yanbin Wang

**Affiliations:** 1 Key Laboratory of Biotechnology and Bioengineering of State Ethnic Affairs Commission, Biomedical Research Center, Northwest Minzu University, Lanzhou 730030, China; 2 College of Life Science and Engineering, Northwest Minzu University, Lanzhou 730030, China; 3 Gansu Tech Innovation Center of Animal Cell, Northwest Minzu University, Lanzhou 730030, China; 4 Faculty of Chemical and Process Engineering Technology, Universiti Malaysia Pahang Al-Sultan Abdullah, Labuh Persiaran Tun Khalil Yaakob, Kuantan 26300, Pahang, Malaysia; 5 Department of Chemical and Process Engineering, Faculty of Engineering and Built Environment, Universiti Kebangsaan Malaysia, Bangi 43600, Selangor, Malaysia; 6 Research Center for Sustainable Process Technology (CESPRO), Faculty of Engineering and Built Environment, Universiti Kebangsaan Malaysia, Bangi 43600, Selangor, Malaysia; 7 Chemical Engineering Institute, Northwest Minzu University, No. 1, Xibeixincun, Lanzhou 730030, China; 8 Key Laboratory of Environment-Friendly Composite Materials of the State Ethnic Affairs Commission, Northwest Minzu University, No. 1, Xibeixincun, Lanzhou 730030, China

**Keywords:** steam flash explosion, keratin nanoparticles, yak horn, structural characterization, biocompatibility

## Abstract

Yak horn represents a valuable resource for fabricating keratin-derived materials; however, its dense, highly cross-linked protein network resists efficient processing via conventional extraction methods. This study introduces a single-step steam flash explosion (SFE) approach to produce keratin nanoparticles (KNPs) from yak horn. The impact of SFE operating pressure (0–1.6 MPa) on KNP morphology, protein secondary/tertiary structures, thermal behavior, and in vitro biocompatibility was systematically investigated. Morphological evaluations revealed that SFE pressure successfully regulates particle dimensions, yielding optimal uniformity with a minimum average particle height of 11.5 nm at 1.45 MPa. Structural characterization indicated that high-pressure treatment induced a shift in disulfide bonds from stable *gauche-gauche-gauche* (*g-g-g*) states (515 cm^−1^) toward higher-energy *trans-gauche-trans* (*t-g-t*) arrangements (540 cm^−1^). This structural deconstruction led to a decrease in thermal degradation temperatures (from 321.9 °C at 0 MPa to <300 °C at 1.6 MPa) but significantly enhanced dehydration efficiency (ΔH = −470.44 J/g at 1.6 MPa). In vitro biocompatibility assessments demonstrated that the prepared KNPs maintain excellent cytocompatibility, supporting L929 and HaCaT cell viabilities above 95%. These findings demonstrated the versatility and effectiveness of SFE as a sustainable strategy for tuning KNP properties, highlighting its great potential in biomedical applications and green material processing.

## 1. Introduction

Keratin is a highly abundant fibrous structural protein characterized by an exceptionally rich cystine content [1]. Depending on the degree of sulfur-mediated cross-linking, native keratin is classified into soft and hard types. Hard keratin, predominantly sourced from animal hooves, wool, feathers, and horns, possesses an abundance of inter- and intra-molecular disulfide bonds that form a dense, mechanically robust, and chemically durable structural matrix [2]. Owing to its excellent mechanical stability, natural biodegradability, low immunogenicity, and intrinsic biocompatibility, keratin has attracted substantial interest across biomedicine, environmental engineering, and cosmetics [3,4,5,6,7].

However, the structural toughness of hard keratin presents a major barrier to its efficient extraction and utilization [2]. Conventional pretreatment via mechanical grinding typically fails to achieve homogeneous matrix refinement, limiting the yield and scalability of downstream nanomaterial processing [8,9,10]. Traditional chemical extraction methodologies, such as aggressive acid/alkaline hydrolysis or oxidative treatments, heavily rely on hazardous reagents, suffer from prolonged operational timelines, and cause severe amino acid degradation, including the irreversible loss of vital tryptophan residues [11]. Enzymatic strategies, though theoretically sustainable, necessitate strict reducing environments for disulfide bond cleavage and suffer from the inherent instability of keratinases under varying temperature and pH [2]. Ionic liquid-based methods, despite their selectivity, face scalability challenges due to high cost and energy-intensive recycling protocols, further constrained by the need for specialized regeneration infrastructure [12]. Microwave-assisted extraction, while enhancing thermal efficiency, may, under inappropriate irradiation conditions, cause protein oxidation or peptide chain scission, thereby compromising product integrity [13]. Collectively, these limitations underscore the urgent need for greener, more efficient, and scalable strategies for keratin-processing and keratin nanoparticle (KNP) production.

Steam flash explosion (SFE) is a promising green thermomechanical technique for biomass processing and nanoparticle fabrication [14]. The process is characterized as an adiabatic expansion, wherein thermal energy is converted into mechanical energy [15]. The sudden energy release causes the superheated water within the material to vaporize instantaneously, generating intense mechanical shear forces that destroy the ordered structure of biopolymers [16]. By adjusting steam pressure and holding time, the particle size of processed biomaterials can be controlled within a specific range, which facilitates the subsequent self-assembly behavior [17]. The SFE method is mainly carried out in a spray explosion device consisting of a cylinder and piston, and the explosion can be completed within 0.0875 s [18]. Previous studies have proven that the SFE method can improve biomass conversion yields compared with conventional treatment methods and has been effectively applied to lignocellulosic biomass, chitin, and keratin [19,20,21].

Although the SFE method has been used to process soft keratin substrates such as feathers, hair and skin, studies on the extraction and utilization of keratin from yak horns remain scarce. Given that the SFE method can disintegrate structurally dense biomacromolecules while maintaining the material’s structural integrity [22,23,24], it could be an effective strategy to process the highly crosslinked keratin structure of yak horns [25]. The successful processing of rigid biomacromolecules, like cellulose (rich in hydrogen-bonded fibrils) and chitin (with crystalline β-linkages), suggests that the SFE method may also be suitable for tough keratin substrates. Therefore, we hypothesized that the highly cross-linked protein network of yak horns could be disrupted by the SFE method and subsequently self-assemble into uniform KNPs under high-pressure steam shock.

This study explored the potential of the SFE method to process structurally robust yak horns and induce keratin self-assembly into uniform nanoparticles while preserving their protein structures. The resulting KNPs were characterized with respect to their physicochemical properties and evaluated for biocompatibility. This work introduces a sustainable SFE-based fabrication route for hard keratin nanomaterials, with potential applications in biomedical and industrial fields.

## 2. Materials and Methods

### 2.1. Materials and Chemicals

Yak horn was purchased from black yak farms in Hezuo, Gannan Tibetan Autonomous Prefecture, China, while phosphate buffer, sodium dodecyl sulfate, potassium persulfate, ethanol, urea, sodium hydroxide, and hexane were purchased from Shanghai Maclin Biochemical Technology Co., Ltd. (Shanghai, China). All commercial chemical reagents were of analytical grade and used as received from the suppliers without further purification.

### 2.2. Sample Collection

The difficulty of the pretreatment stage was increased by the extreme toughness and elastic strength of yak horn. For powder preparation, considering that the denaturation temperature of keratin is more than 100 °C [26], the medullary part of yak horn was first removed using the steaming method to obtain the fibrous shell. The fibrous shell was then milled to the particle size suitable for the SFE equipment. The dried yak horn powder was sieved through a 325-mesh screen to standardize the baseline particle size distribution below 45 μm. To eliminate lipids and cellular debris that could alter downstream self-assembly, the sieved powder was transferred into a Buchi B-800 extractor and subjected to continuous hot Soxhlet extraction using analytical grade n-hexane at 65 °C for 8 h. Following degreasing, the matrix was demineralized by immersion in 0.1 M sodium phosphate buffer (pH 7.4) for 24 h to leach out calcium phosphate and residual inorganic mineral salts. The refined slurry was rinsed repeatedly with double-distilled water until the supernatant reached a neutral pH of 7.0 ± 0.1. The resulting purified, defatted yak horn powder was vacuum-dried at 60 °C for 24 h to achieve a constant moisture content below 5 wt%.

### 2.3. Steam Flash Explosion Treatment

**Figure 1:**
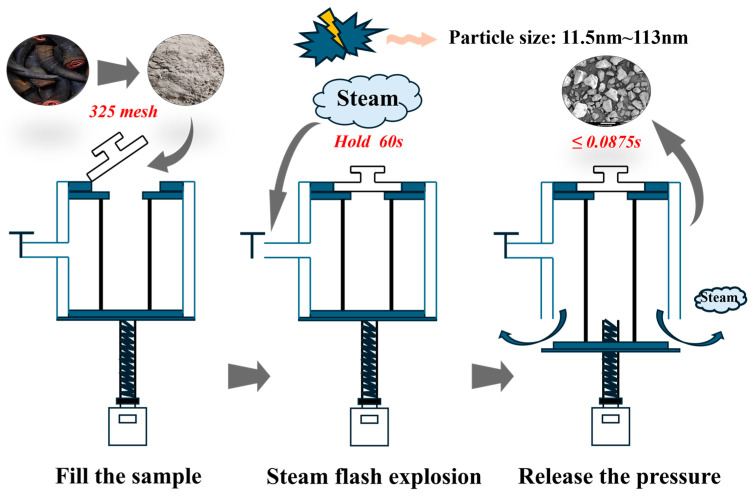
Schematic diagram of the steam flash explosion treatment process.

The SFE treatment, specifically tailored for yak horn, was conducted using a QB-300B model from Qingfeng Eco-technology Co., Ltd. (Suzhou, China). The experimental groups were subjected to four discrete, pre-set steam pressures: 0.45, 1.00, 1.45, and 1.60 MPa, representing calculated saturation steam temperatures of approximately 148, 180, 198, and 201 °C, respectively. For each execution cycle, a calibrated mass of 100 g of the pre-conditioned yak horn powder was loaded into the 5 L sealed reaction chamber of the apparatus. As shown in Figure 1, high-pressure saturated steam was rapidly injected until the target pressure was reached, followed by a strict equilibration holding duration of 60 s to guarantee uniform thermodynamic penetration across the biomass granules. The pneumatic actuation valve was then automatically tripped, completing the sudden explosive decompression into a receiver cyclone within an instantaneous burst speed of 0.0875 s [27]. After steam flash explosion, the obtained samples were collected, washed with deionized water, freeze-dried, and subsequently used for multi-scale structural characterization.

### 2.4. Scanning Electron Microscopy (SEM)

A small amount of sample powder was dispersed onto conductive adhesive tape mounted on the sample stub, purged with a washer ball and sputter-coated with gold for 60 s. The surface morphology of the sample was then examined by scanning electron microscopy (SEM; SU8220, Hitachi High-Tech Corporation, Tokyo, Japan) in secondary electron imaging mode, at a standard beam current, and at an accelerating voltage of 10 kV [28].

### 2.5. Atomic Force Microscopy (AFM)

Surface morphology and sample thickness were further analyzed using an atomic force microscope (AFM; MultiMode 8 Nanoscope, Bruker Corporation, Billerica, MA, USA). The samples were scanned using a silicon nitride cantilever with a nominal tip radius of 10 nm at 25 °C. Imaging was performed in tapping mode at a scan frequency of 50 Hz and a scan rate of 0.8 Hz [29].

### 2.6. Fourier Transform Infrared Spectroscopy (FTIR)

The samples were characterized by Fourier transform infrared spectroscopy (FTIR; Nicolet iS10, Thermo Fisher Scientific, Madison, WI, USA) to identify their functional groups. A small amount of sample was mixed with KBr at a mass ratio of 1:10–1:20 using a mortar and pestle. The mixture was then pressed into a pellet for analysis. A blank KBr pellet was used as the background. The spectra were recorded over the range of 4000–400 cm^−1^ at a resolution of 4 cm^−1^ with 32 scans.

### 2.7. Raman Spectroscopy

Raman spectra were measured using the inVia-Reflex Raman Microscope (Renishaw plc, Gloucestershire, UK) over the spectral range of 4000–100 cm^−1^. The keratin samples were first dried at 50 °C for 5 h before the analysis. The spectral measurements were repeated independently three times for all samples, with 100 accumulations for each spectrum. A 100×/0.75 objective lens was used for sample focusing, providing a spatial resolution of approximately 20 μm. The Raman signal was collected in reflection mode using an f/1 collection lens.

### 2.8. Thermogravimetric Analysis (TGA)

Several milligrams of dried sample were placed evenly in a TGA crucible and analyzed using a thermogravimetric analyzer (TG209F3 Tarsus, NETZSCH-Gerätebau GmbH, Selb, Germany) under a nitrogen atmosphere. Before measurement, the furnace was purged with nitrogen to remove residual air. The samples were heated from 25 °C to 600 °C at a heating rate of 10 °C/min under a nitrogen flow rate of 50 mL/min.

### 2.9. Differential Scanning Calorimetry (DSC)

Approximately 5–10 mg of dried sample was placed evenly in a DSC crucible and analyzed using a differential scanning calorimeter (Q20, TA Instruments, New Castle, DE, USA) under a nitrogen atmosphere. Before measurement, the furnace was purged with nitrogen to remove residual air. The samples were heated from 25 °C to 600 °C at a heating rate of 10 °C/min under a nitrogen flow rate of 50 mL/min.

### 2.10. Cytotoxicity

The in vitro cytotoxicity and cell proliferation profiles of the prepared KNPs were systematically evaluated using both mouse fibroblast cells (L929) and human immortalized keratinocytes (HaCaT). Both cell lines were purchased from Procell Life Science & Technology Co., Ltd. (Wuhan, China). The cells were cultured in Dulbecco’s Modified Eagle Medium (DMEM) supplemented with 10% fetal bovine serum (FBS) and 1% penicillin–streptomycin, and maintained in a humidified incubator at 37 °C with 5% CO_2_. L929 cells were seeded into 96-well plates at a density of 5 × 10^3^ cells per well. After 12 h of incubation, keratin samples treated with SFE under different pressures were added to the culture medium, and the cells were further cultured for 1 and 3 days. To conduct a rigorous high-dose limit test (worst-case scenario screening) for the physical deconstruction process, the KNP extract fluid was prepared at a standardized, maximized screening concentration of 10 mg/mL. Briefly, 0.1 g of sterile KNP powder from each pressure group was immersed in 10 mL of serum-free DMEM culture medium and incubated at 37 °C for 24 h under continuous agitation. After incubation, the crude extract was subjected to high-speed centrifugation (10,000 rpm for 15 min) and subsequently passed through a 0.22 μm sterile syringe filter to thoroughly eliminate any suspended particulate debris or optical interference noise. Prior to cell treatment, the clarified stock extract was supplemented with 10% FBS and 1% penicillin–streptomycin to obtain the final 100% complete extraction medium. For the cytocompatibility assay, both L929 and HaCaT cells were seeded into 96-well plates at a density of 5 × 10^3^ cells per well. After 12 h of initial incubation to allow cell attachment, the original culture medium was replaced with the prepared KNP complete extraction medium, and the cells were further cultured for 1 and 3 days. Cell viability was evaluated using the Cell Counting Kit-8 (CCK-8) assay. Live/dead staining was also performed to visualize cell survival. The cell viability was calculated as follows:

$$Cell\;Viability\;\left(\%\right)=\frac{OD\;of\;Experimental\;group}{OD\;of\;Control\;group}\times 100\%$$

The results from quintuplicate experiments were averaged.

### 2.11. Statistical Analysis

All quantitative experimental data, including nanoparticle dimensions, structural proportions, and cell viability metrics, were collected from at least three independent replicates and are expressed as mean ± standard deviation. Graphical illustrations and statistical evaluations were performed using OriginPro2022 software. Statistical significance among multiple experimental groups was analyzed by one-way analysis of variance (ANOVA), followed by Tukey’s post-hoc test for homogeneous variance pairwise comparisons. A *p*-value of less than 0.05 (*p* < 0.05) was considered statistically significant, whereas *p* > 0.05 was designated as not statistically significant (ns).

## 3. Results

### 3.1. Observation of the Apparent Structure

**Figure 2:**
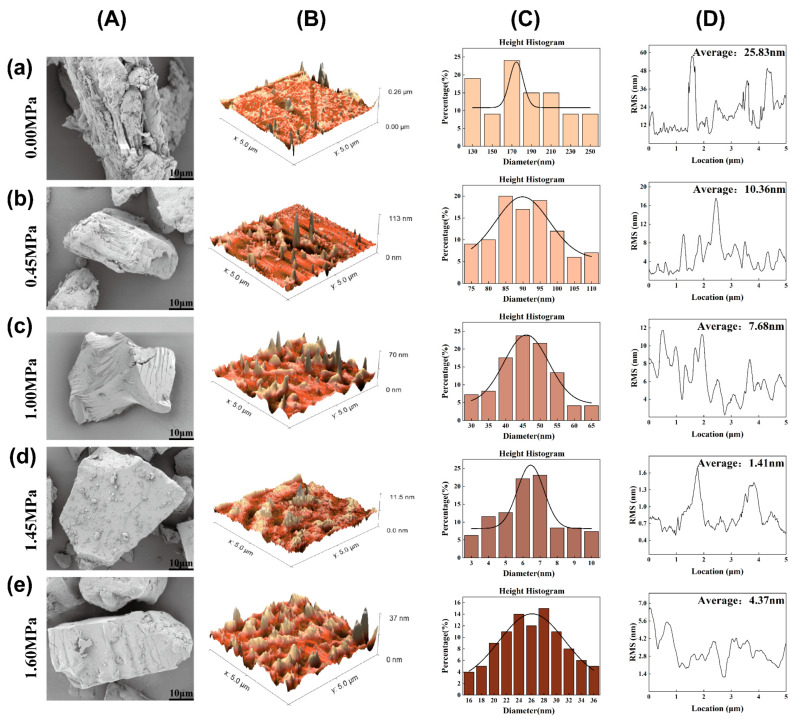
Characterization of keratin samples subjected to various SFE pressures. (**a**–**e**) 0–1.6 MPa; the 0 MPa group represents the untreated yak horn (control), while the remaining groups were treated by steam flash explosion at pressures of 0.45, 1.0, 1.45, and 1.6 MPa, respectively. (**A**) SEM images showing the morphological evolution of keratin structures. (**B**) AFM 3D images highlighting surface topography and roughness changes. (**C**) Particle size distribution histograms of keratin. (**D**) Mean surface roughness (RMS) values derived from AFM measurements.

The structural changes in keratin induced by various SFE pressures were systematically investigated through integrated SEM, AFM, and particle size distribution analyses (Figure 2), which revealed controlled pressure-dependent disintegration [30] and reorganization mechanisms of keratin architecture during high-pressure processing [31,32]. Tapping mode atomic force microscopy can accurately measure the vertical height of individual particles without interference from the tip broadening effect, which is a reliable method for size characterization of soft protein nanoparticles [29].

The morphological evolution of the processed keratin under different SFE pressure thresholds was systematically monitored via SEM (Figure 2A(a–e)) and AFM (Figure 2B). In the dry-state dry-powder SEM images, the materials across all pressure groups exhibit micro-scale clustered blocks. This macro-appearance is attributed to the high surface energy and extensive exposed hydrophilic networks of the refined building blocks, which naturally drive kinetic secondary self-assembly and hierarchical aggregation during the freeze-drying phase. To successfully eliminate aggregation noise and uncover the true dimensions of the primary structural units, tapping-mode AFM was conducted on highly diluted dispersions. With the initial step up in SFE pressure to 0.45 MPa, the dense matrix began to loosen and disintegrate, yielding primary structural building blocks with an average vertical height of 113 nm. At 1.45 MPa (Figure 2A–C(d)), the keratin sample was present in the form of uniform nanoscale particles, with the average particle height decreasing to 11.5 nm. Combined with the particle size distribution statistics (Figure 2C), it can be seen that the particle size distribution under this condition is relatively concentrated, showing good uniformity. This observation confirms the successful fabrication of KNPs. A further increase in pressure to 1.6 MPa resulted in a slight increase in particle height to 36.7 nm, likely due to overexposure to mechanical and thermal energy during SFE treatment [33,34]. This underscores the importance of carefully optimizing SFE conditions to avoid over-processing, which may adversely affect KNP uniformity and functionality [30,34].

In addition to the height and diameter distributions, the micro-topographic variations across different processing windows were quantitatively analyzed using the root-mean-square roughness (RMS) profiles derived from AFM measurements (Figure 2D). The untreated 0 MPa control displayed the highest roughness (25.83 nm). SFE treatment progressively reduced the RMS to 10.36 nm at 0.45 MPa and 7.68 nm at 1.00 MPa, confirming effective thermomechanical disruption of the dense matrix. Crucially, the RMS reached its minimum (1.41 nm) at the optimized pressure of 1.45 MPa, indicating thorough exfoliation into ultra-fine, well-dispersed primary building blocks. However, further increasing the pressure to 1.60 MPa caused the RMS to rebound to 4.37 nm. This topographic deterioration confirms that over-processing at excessive thermodynamic thresholds drives protein chain unfolding and secondary self-aggregation, thereby disrupting structural homogeneity and aligning perfectly with the height and diameter fluctuations captured upstream.

### 3.2. Fourier Transform Infrared Spectroscopy (FTIR) Analysis

**Figure 3:**
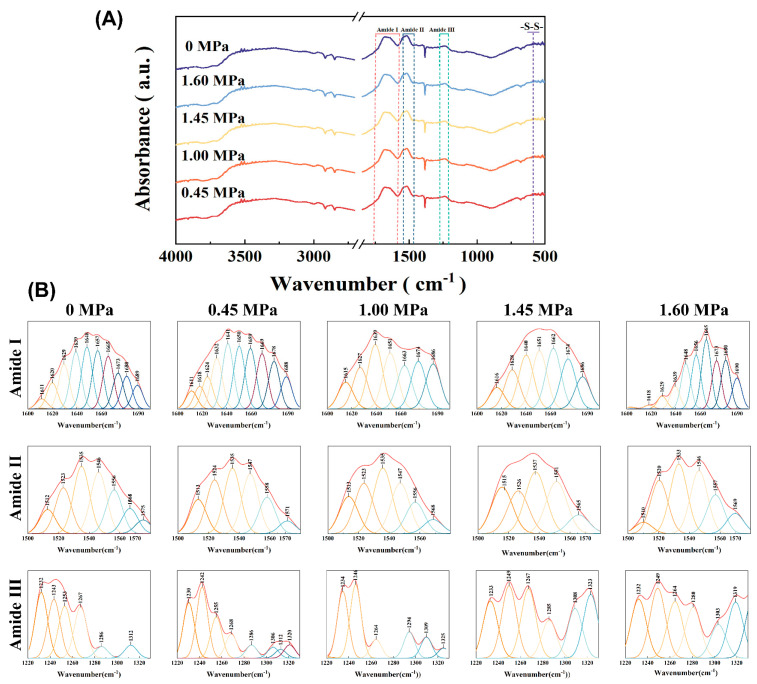
(**A**) FTIR spectra of keratin samples treated under various SFE pressures (0 MPa–1.6 MPa), including Amide I (1700–1600 cm^−1^), Amide II (1600–1500 cm^−1^), Amide III (1300–1200 cm^−1^), and the disulfide bonds (600–500 cm^−1^). (**B**) Peak fitting of the Amide I (1700–1600 cm^−1^) and Amide III (1330–1220 cm^−1^) regions from the FTIR spectra.

The secondary structure of KNPs during SFE was characterized using FTIR. Absorption bands at around amide I (1700–1600 cm^−1^, C=O stretching vibrations), amide II (1600–1500 cm^−1^, N–H bending vibrations or C–N stretching vibration), amide III (1330–1220 cm^−1^, C–O and C–N stretching, O=C–N and N–H bending), and the disulfide bonds (600–500 cm^−1^) were analyzed to evaluate secondary structural changes in keratin [18,35]. After SFE treatment, the KNPs showed no new characteristic peaks (Figure 3A), indicating that SFE treatment did not induce the formation of additional functional groups.

**Figure 4:**
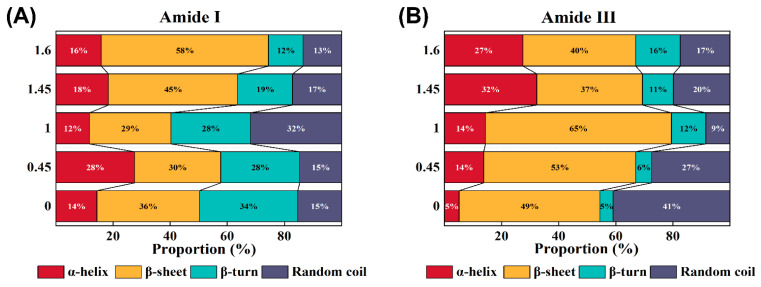
Deconvolution and quantitative analysis of the (**A**) Amide I (1700–1600 cm^−1^) and (**B**) Amide III (1330–1220 cm^−1^) bands from FTIR spectra of keratin samples treated under various SFE pressures (0–1.6 MPa).

In addition, the amide bands of the KNPs were subjected to peak fitting (Figure 3B) and semi-quantitative analysis (Figure 4) to evaluate pressure-induced changes in protein secondary structure [36]. The proportion of β-sheet structures initially decreased from 36% to 29% and subsequently increased to 58% with increasing SFE pressure (Figure 4A). The non-monotonic variation in β-sheet content reflects a distinct deconstruction–aggregation pathway driven by SFE thermodynamic stress. The initial decrease from 36% to 29% is attributed to the disruption of native hydrogen bonding networks within the crystalline domains of hard keratin, driven by superheated steam infiltration and intense physical shear [31]. This initial thermomechanical deconstruction forces the rigidly cross-linked structural matrix to temporarily unfold and loosen. Conversely, the sharp surge to 58% at 1.60 MPa is governed by thermal- and shear-induced kinetic self-assembly. Exceeding this critical thermodynamic energy threshold triggers extensive peptide chain unfolding, which exposes previously buried internal hydrophobic domains. Under excessive physical stress and subsequent decompression cooling, these high-energy unfolded domains undergo rapid re-association, driving the extensive formation of highly compacted frameworks, which is in excellent agreement with the findings of Nakagawa et al. [37]. The initial reduction suggests that moderate SFE treatment disrupted the intermolecular hydrogen-bonding network within the native keratin, resulting in partial unfolding of the ordered β-sheet domains. As the explosion pressure increased further, the unfolded peptide chains underwent structural rearrangement and re-association during rapid decompression, promoting the formation of more compact β-sheet structures. This pressure-dependent unfolding–refolding behavior has also been reported in thermomechanically treated protein materials [33,38]. The Amide II Band (1600–1500 cm^−1^) arises from N–H bending and C–N stretching vibrations in the protein backbone [39,40] and exhibited slight intensity reductions at higher pressure without significant band shifts. The Amide III band (1330–1220 cm^−1^) complements the Amide I region in reflecting secondary structural changes, particularly those involving β-sheets [41]. The β-turn proportion increased from 0 to 1.6 MPa (Figure 4B), suggesting that SFE treatment may have initially induced the formation of β-turns, which could serve as intermediates in the structural transition towards β-sheets [42,43,44].

### 3.3. Raman Spectroscopy Analysis

**Figure 5:**
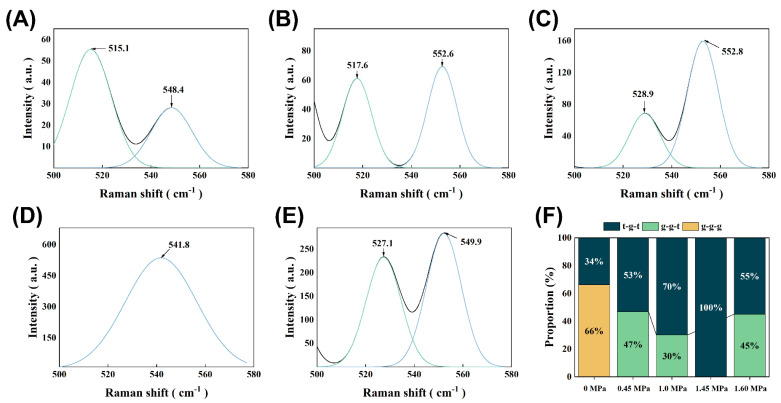
Raman spectroscopy analysis of yak horn keratin treated at different SFE pressures (0–1.6 MPa) (**A**–**E**). (**F**) Disulfide bond content analysis showing the relative intensity of *t-g-t*, *g-g-t*, and *g-g-g* conformations under different pressure treatments.

Raman spectroscopy was used to analyse the three basic conformations of disulfide bonds related to the tertiary structure of keratin. The untreated yak horn keratin (0 MPa, Figure 5A) displayed characteristic peaks in the range of 500–580 cm^−1^. The peak at 515 cm^−1^ is associated with the *gauche-gauche-gauche* (*g-g-g*) disulfide bond conformation. This conformation was commonly observed in stable, native keratin structures [45]. At 1.45 MPa (Figure 5D), the *trans-gauche-trans* (*t-g-t*) conformation became dominant, with a characteristic peak at 541.8 cm^−1^ (Figure 5F), while *gauche-gauche-trans* (*g-g-t*) and *t-g-t* peaks were observed at 527.1 cm^−1^ and 549.9 cm^−1^ (Figure 5E), respectively. The higher wavenumber of the *t-g-t* conformation reflects a relatively higher energy state, which may imply reduced structural stability under high-pressure conditions [24]. This structural alteration is likely related to the mechanical properties and biological functions of keratin [23]. At 1.6 MPa, the *g-g-g* conformation was no longer detected, indicating that the disulfide bonds predominantly adopted the *t-g-t* (55%) and *g-g-t* (45%) conformations under the highest applied pressure (Figure 5F). This phenomenon may be attributed to the rapid movement of high-pressure vapour and the instantaneous release of mechanical shear, which may cause disulfide bonds in the *t-g-t* conformation to become more prone to fracture or rearrangement, thereby increasing their rotational freedom and leading to conformational changes [16].

### 3.4. Thermogravimetric Analysis (TGA)

**Figure 6:**
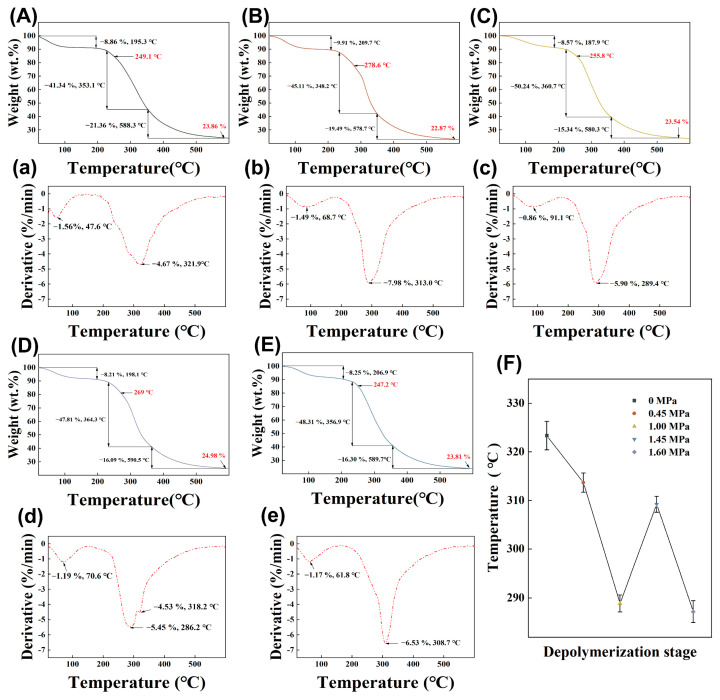
Thermal stability and degradation behavior of yak horn keratin samples processed under various SFE pressures (0–1.6 MPa). (**A**–**E**) Thermogravimetric analysis (TGA) curves of KNPs treated at 0–1.6 MPa. (**a**–**e**) Derivative thermogravimetric (DTG) curves corresponding to (**A**–**E**). (**F**) Comparative summary of the highest temperature of the depolymerization stage across all pressure groups.

Thermogravimetric analysis (TGA) curves (Figure 6A–E) provide comprehensive insights into the overall thermal stability and distinct mass loss stages of yak horn KNPs processed under different SFE pressures (0–1.6 MPa), while the corresponding derivative thermogravimetric (DTG) curves (Figure 6a–e) yield precise numerical data regarding the degradation kinetics and maximum decomposition rates. To further quantify these thermal behaviors, key characteristic parameters, including the temperature at 10% weight loss (*T*_10%_) and the final residual mass at 600 °C, are summarized in Table 1.

The thermograms were divided into three distinct thermal degradation phases, consistent with previous reports [5]. In the first phase, which involved dehydration and loss of volatile substances, no significant differences were observed among samples treated at different pressures. The mass loss in this stage ranged from 8.21% to 9.91% (Figure 6A–E). In the second phase, corresponding to the primary decomposition stage, the covalent peptide bonds within amino acid residues underwent cleavage [5]. Notably, samples treated at 0 MPa exhibited the highest decomposition temperature (*T_max_*) of 321.9 °C (Figure 6a) and the lowest mass loss of 41.34% during this stage (Figure 6A). This phenomenon may be attributed to the stable native keratin structure, in which non-covalent interactions and hydrogen bonds remained intact [16,38]. As the SFE pressure was altered, the maximum depolymerization temperatures shifted accordingly, as summarized in the comparative profiling (Figure 6F). In the third phase, further decomposition of protein molecules occurred and was associated with the disruption of β-sheet conformations and the cleavage of disulfide bonds [46,47]. In this phase, no significant differences in residual mass were observed among the groups, with the residual mass ranging from 22.87% to 24.98% (Figure 6A–E).

### 3.5. Differential Scanning Calorimetry (DSC)

**Figure 7:**
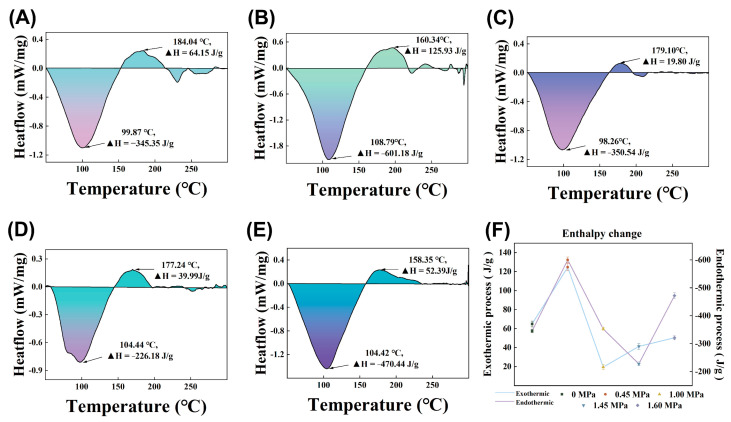
DSC curves of KNPs under different SFE pressures (0–1.6 MPa). (**A**–**E**) DSC data at SFE pressures of 0, 0.45, 1.00, 1.45, and 1.6 MPa. (**F**) Summary of DSC data showing the exothermic process and endothermic process at various pressures.

As illustrated in Figure 7, a broad endothermic peak was observed near 100 °C across all pressure groups. In alignment with the established thermal characterization of hard biopolymers, this event is primarily attributed to the forced desorption of structurally bound water tightly locked within the crystalline domains of keratin, closely coupled with the cooperative disruption of the intra-molecular hydrogen-bonding networks [48]. As the pressure increased, the absolute value of the enthalpy of vaporization also increased, reaching ΔH = −470.44 J/g at 1.6 MPa. This trend indicates that higher SFE pressures induced more pronounced conformational changes and exposed a greater abundance of hydrophilic residues, thereby increasing the density and binding affinity of the structural water within the KNP matrix.

In the subsequent thermal transition phase, the exothermic peak temperature exhibited a non-monotonic trend, first increasing and then decreasing with rising pressure. The 0 MPa control group displayed a distinct exothermic event at 184.04 °C with ΔH = 64.15 J/g. This behavior suggests that while moderate SFE processing can optimize the structural ordering and thermal stability of the proteins, excessively high pressures tend to compromise this stability. Furthermore, the reduction in exothermic enthalpy following SFE treatment (declining to 52.39 J/g) demonstrates that less energy was required for the structural unfolding of the treated samples. This phenomenon was driven by the initial destabilization of intra-molecular bonds by the intense shear forces and cavitation effects generated during the SFE decompression process [16].

### 3.6. In Vitro Cytocompatibility Evaluation

**Figure 8:**
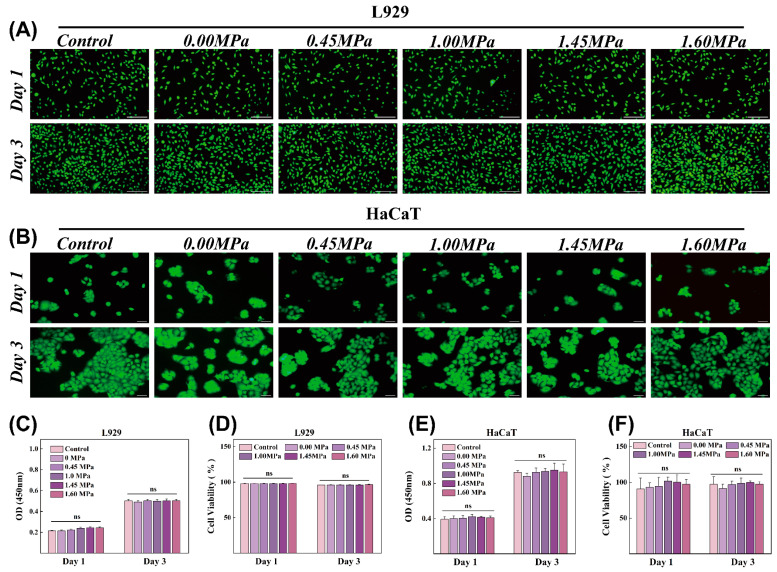
In vitro cytocompatibility evaluations of L929 mouse fibroblasts and HaCaT human keratinocytes treated with SFE-processed KNPs. (**A**) Live/dead fluorescence staining micrographs of L929 cells after 3 days of incubation, and (**B**) HaCaT cells after 3 days of incubation (scale bars = 200 μm). (**C**,**D**) Quantitative cell metabolic activity and viability of L929 cells, and (**E**,**F**) HaCaT cells determined by CCK-8 assays after 1 and 3 days of continuous exposure. All quantitative data are presented as mean ± standard deviation derived from four independent biological replicates (*n* = 4), while *ns* indicates not statistically significant (*p* > 0.05).

The cytocompatibility of yak horn keratin obtained under different SFE pressures was evaluated using L929 fibroblasts and HaCaT keratinocytes (Figure 8). Live/dead staining revealed predominantly green fluorescence in all groups after both 1 and 3 days of culture, indicating that the keratin extracts did not induce detectable cytotoxic effects (Figure 8A,B). Furthermore, the cell density increased markedly from Day 1 to Day 3 for both cell lines, suggesting sustained cell proliferation during the culture period. These observations were further supported by the CCK-8 assay, in which the OD450 values increased significantly over time in all groups (Figure 8C,E). However, no significant differences were observed among the control and pressure-treated samples at either time point (*p* > 0.05). Similarly, quantitative viability analysis demonstrated that the viability of both L929 and HaCaT cells remained above 95% throughout the experimental period, with no statistically significant differences among groups (Figure 8D,F). These results indicate that SFE treatment, despite inducing substantial alterations in keratin morphology and molecular structure, did not compromise its biological compatibility. Previous structural analyses demonstrated that SFE treatment promoted disulfide bond rearrangement, modified crystalline organization, and altered thermal behavior of keratin; however, these pressure-induced structural transformations did not adversely affect cellular responses. The consistently high viability and proliferation rates observed for both fibroblasts and keratinocytes suggest that the intrinsic biocompatibility of keratin was well preserved after SFE processing. Therefore, SFE can be considered an effective and environmentally friendly strategy for the valorization of yak horn keratin while maintaining excellent cytocompatibility, supporting its potential application in future biomedical and tissue engineering fields [49,50].

## 4. Discussion

### 4.1. Thermomechanical Disintegration and Morphological Evolution

The top-down deconstruction of the structurally dense yak horn matrix into uniform KNPs underscores the unique thermodynamic advantages of SFE. The complete absence of fragmentation observed in the untreated 0 MPa control group confirms that conventional mechanical grinding is insufficient for the homogeneous refinement of hard keratins. This limitation arises from the high density of inter-chain disulfide cross-links and the structural anisotropy characteristic of bovine horn matrices [51]. The transition to highly uniform nanoparticles with a minimized average height of 11.5 nm at 1.45 MPa demonstrates that the rapid adiabatic expansion of superheated water vapor generates intense mechanical shear and cavitation forces capable of overcoming the structural cohesion of the native keratin aggregates [52]. However, the counter-undulation in particle height to 36.7 nm observed when the pressure was increased to 1.6 MPa marks a distinct operating threshold. Exceeding this critical pressure introduces excessive thermal and mechanical energy into the system [53]. This over-processing induces extensive unfolding of the protein chains, which increases the exposure of internal hydrophobic domains and drives kinetic secondary self-aggregation during the explosive decompression phase. This phenomenon is consistent with the findings of Hu et al. and Vadillo et al. [30,33], confirming that optimizing the thermodynamic processing window is essential to maintain nanoparticle uniformity and prevent thermal aggregation during high-pressure biopolymer processing.

Similar morphological evolution has also been reported in previous studies on steam explosion-assisted keratin extraction, although most investigations focused on relatively soft keratin resources such as feathers and wool. Tonin et al. [21] demonstrated that steam explosion effectively disrupted the compact fibrous architecture of wool keratin, resulting in increased structural accessibility without severe chemical degradation. Likewise, Zhang et al. [18] reported that steam flash explosion facilitated the dissolution of feather keratin by breaking intermolecular interactions while largely preserving the protein backbone. Compared with these relatively soft keratin sources, yak horn possesses a considerably denser hierarchical organization with abundant disulfide cross-links and a higher degree of structural anisotropy, making its thermomechanical deconstruction substantially more challenging. Therefore, the successful generation of nanoscale keratin particles in the present study demonstrates that SFE is applicable not only to soft keratin but also to highly cross-linked hard keratin matrices, thereby extending the processing scope of this environmentally friendly technology.

### 4.2. Concomitant Conformational Rearrangements of Secondary and Tertiary Structures

The multi-scale structural transformations monitored via FTIR and Raman spectroscopy elucidate the underlying mechanism of SFE-induced keratin deconstruction. The preservation of the baseline Amide I, II, and III vibration bands across all treated groups confirms that the instantaneous explosion process modifies the spatial conformation of the protein without disrupting its fundamental chemical composition or introducing extraneous functional groups.

The variation in β-sheet content (decreasing from 36% to 29% before rising to 58% at 1.6 MPa) reflects a distinct denaturation–compaction pathway. Initial SFE pressures disrupt the weaker inter- and intra-molecular hydrogen bonding networks within the crystalline domains of native keratin, causing temporary protein unfolding [21]. As the explosion pressure climbs toward 1.6 MPa, the synergistic effect of superheated steam molecules and high kinetic energy forces the unfolded peptide chains to undergo rapid re-association, condensing into highly compacted, tightly arranged β-sheet structures. This non-monotonic denaturation–compaction pathway differs significantly from traditional chemical extraction or aggressive acid/alkali hydrolysis. Conventional harsh chemical treatments usually cause irreversible degradation of the protein backbone, leading to a permanent collapse of ordered crystalline domains into disordered random coils [54]. In contrast, the physical thermomechanical stress of SFE drives a controlled unfolding–refolding transition, allowing the loosened chains to self-assemble into a more compact, highly ordered crystalline network.

The continuous accumulation of β-turn fractions further suggests that these specific conformations act as stable structural intermediates, accommodating the sharp chain turns required during the rapid transition from random coils back to ordered β-sheets.

On a tertiary structural level, the complete disappearance of the native low-energy *gauche-gauche-gauche* (*g-g-g*) disulfide peak (515 cm^−1^) and its subsequent shift toward the higher-energy *trans-gauche-trans* (*t-g-t*) conformation (541.8–549.9 cm^−1^) prove that SFE successfully overcomes the covalent energy barriers of the hard keratin matrix. The rapid movement of high-pressure vapor and instantaneous decompression provide the necessary activation energy to induce torsional rotation around the S–S and C–S bonds, forcing the disulfide bridges into higher-energy, more flexible configurations [55,56].

### 4.3. Thermodynamic Interplay and Thermal Behavior Trade-Offs

The quantitative insights obtained from TGA and DSC analyses reveal a clear correlation with the observed structural rearrangements. In the primary decomposition phase, the downward shift of the thermal degradation temperature below 300 °C for the SFE-treated samples compared to the untreated control (321.9 °C) initially suggests a reduction in thermal stability. This thermal behavior implies a potential correlation with the multi-scale conformational transition metrics captured by our upstream characterization. It is highly expected that the extensive stretching of covalent disulfide linkages from native states into the higher-energy *t-g-t* configurations introduces a distinctive internal structural tension. This space-conforming alteration subsequently breaks the protective native hydrogen-bonding networks that stabilize the crystalline domains of hard keratin [57]. Such structural loosening significantly reduces the energy barrier required for thermal pyrolysis, thereby accelerating the primary peptide backbone cleavage and causing it to occur at lower onset temperatures [46]. This systematic downward shift in the thermal decomposition threshold aligns with the thermodynamic behaviors observed in other flash-exploded or intensively milled biopolymers. Previous studies on the steam explosion of structural proteins have firmly established that the rapid disruption of dense crystalline regions and the loss of molecular entanglement invariably lower the thermal stability onset [18]. This similarity confirms that the intense physical stress of SFE successfully undermines the native thermal resistance of the dense yak horn matrix.

Paradoxically, while the initial thermal decomposition threshold decreases, the bound-water vaporization enthalpy (Δ*H*) captured during DSC scanning climbs significantly, reaching a maximum of −470.44 J/g at 1.6 MPa. This thermal trade-off reflects the structural changes associated with nanostructural conversion. The deconstruction of dense, bulk yak horn aggregates into ultra-small nanoparticles exponentially expands the total specific surface area of the protein. This morphological transition forces previously buried polar, hydrophilic functional groups (such as -COOH and -NH_2_) to orient outward toward the nanoparticle–fluid interface. These newly exposed hydrophilic sites form extensive networks of bound water molecules via hydrogen bonding [26,58]. Consequently, evaporating this tightly bound water shell requires a substantially higher input of thermal energy, explaining the significantly elevated endothermic enthalpy values recorded during DSC analysis. This sharp elevation in ΔH provides strong thermodynamic proof of successful nanomaterial conversion, closely matching the hydration theories of downsized biopolymers. According to classic thermal analysis profiles of hydrated proteins, as biomacromolecules are refined from macro-aggregates into nanoscale domains, the exponential surge in exposed surface functional sites drastically increases the fraction of tightly bound water [59]. The high vaporization energy recorded in our 1.6 MPa group demonstrates that SFE serves as an exceptionally efficient thermodynamic tool to unlock and scale up the hidden hydrophilic capacities of hard keratin.

### 4.4. In Vitro Biological Safety and Structure–Function Compatibility

Evaluating cellular responses is critical to determine whether the aggressive thermomechanical shear of SFE compromises the biological safety of the processed protein. Quantitative metabolic metrics derived from four independent biological replicates (*n* = 4) demonstrated that continuous exposure to KNPs across all pressure groups induced no statistically significant suppression of cell viability compared to the negative cell control (*p* > 0.05). Both L929 mouse fibroblasts and HaCaT human keratinocytes consistently maintained excellent cell survival rates exceeding 95% over a 3-day culture period. These cells have a healthy, typical spindle-shaped or oval appearance. This excellent cytocompatibility profile highlights the distinct advantages of the SFE manufacturing approach. Traditional chemical extraction methods for hard keratins frequently rely on toxic reducing agents or hazardous organic solvents, which can remain trapped within the regenerated protein matrix as trace residues that cause cytotoxicity in sensitive skin lines [49,60]. In contrast, the SFE process uses superheated water vapor as the sole working fluid and breaking mechanism. Because it avoids any foreign organic chemicals or toxic synthetic cross-linkers, the resulting KNPs are highly pure and completely free of toxic chemical residues. From a structural perspective, the Raman and FTIR data confirm that although SFE processing breaks down the bulk structure and shifts the disulfide bonds into higher-energy states, it mainly causes a non-destructive spatial unfolding of the protein rather than a chemical degradation of its primary sequence. Consequently, the core amino acid sequences responsible for cell–matrix interactions remain functional, allowing excellent cell survival and proliferation.

### 4.5. Limitations and Future Research Directions

This study mainly characterized the morphology and size of KNPs in the dry state by SEM and AFM. The particle dispersion behavior in the hydrated state has not been systematically investigated, nor has the fine internal morphology of particles been further characterized by transmission electron microscopy. Subsequent studies will supplement relevant characterizations to improve the multi-dimensional morphological characterization system of the material.

Although the quantitative physical, chemical, and in vitro biological characterizations establish a consistent structure–property relationship for SFE-derived KNPs, certain limitations should be acknowledged. While the CCK-8 and Calcein-AM fluorescence assays confirmed baseline cytocompatibility against L929 and HaCaT cell lines, the performance of these nanoparticles in application-specific environments remains to be fully verified.

## 5. Conclusions

This study establishes a sustainable, highly efficient, and chemical-free top-down paradigm for fabricating uniform KNPs from highly cross-linked, recalcitrant yak horn matrices via SFE. Through precise thermodynamic tuning, the SFE process successfully shatters the severe steric hindrance and dense covalent cross-linking networks of native hard keratin. Under the optimized operating pressure of 1.45 MPa, the broad, micron-scale aggregates featuring an average height of 260 nm and a root-mean-square roughness of 25.83 nm were effectively exfoliated into ultra-fine, well-dispersed nanoparticles with a minimized average height of 11.5 nm and an ultra-smooth topographic profile of 1.41 nm. Multi-scale structural and conformational fingerprinting elucidates the underlying deconstruction–reorganization mechanisms driven by intense thermomechanical shear and cavitation. The protein matrix undergoes a distinct pressure-dependent denaturation–compaction pathway, wherein the beta-sheet fraction transitions non-monotonically from 36 to 29% before dynamically consolidating into a highly dense crystalline framework of 58% at 1.60 MPa. On the tertiary molecular level, SFE successfully overcomes covalent energy barriers, shifting the disulfide bridges from their native, low-energy gauche-gauche-gauche conformation at 515 cm^−1^ under 0 MPa completely into higher-energy trans-gauche-trans and gauche-gauche-trans networks at 1.60 MPa, which account for 55% and 45% of the total conformations, respectively, thereby imparting enhanced molecular rotational freedom.

Quantitative thermal analysis establishes a robust, highly consistent structure–property relationship linked directly to these conformational rearrangements. The induced structural tension and the loosening of crystalline domains permanently reduce the macroscopic thermal decomposition threshold, shifting the maximum decomposition temperature from 321.9 °C in the native control down to 286.2 °C at 1.60 MPa, which aligns perfectly with a decreased structural unfolding enthalpy from 64.15 J/g to 52.39 J/g. Paradoxically, this top-down nanostructural conversion exponentially expands the specific surface area, forcing previously buried polar functional groups to re-orient outward. This structural re-ordering dramatically intensifies the binding affinity and density of structurally bound water locked within the KNP matrix, demanding an exceptionally elevated forced desorption enthalpy of −470.44 J/g during differential scanning calorimetry scanning. Furthermore, rigorous in vitro biological safety evaluations derived from independent replicates (*n* = 4) validate the superb cytocompatibility of these chemical-free building blocks, supporting both L929 mouse fibroblast and HaCaT human keratinocyte viabilities consistently above 95% over a 3-day culture period with robust proliferation and zero residue-induced cytotoxicity. Collectively, these quantifiable insights position SFE as a milestone green processing technology capable of precisely tailoring the multi-scale physicochemical and thermodynamic traits of hard biomass wastes, establishing these highly pure KNPs as premier candidates for advanced interactive wound dressings, responsive dermal regeneration matrices, and next-generation tissue engineering scaffolds.

## Figures and Tables

**Table 1:** Standard thermal degradation parameters of yak horn KNPs processed under various SFE pressures.

SFE Pressure (MPa)	*T*_10%_ (°C)	*T_max_* (°C)	Residual Weight at 600 °C (%)
0 (Control)	213.9	321.9	23.86
0.45	164.8	313.0	22.87
1.00	213.7	289.4	23.54
1.45	210.5	308.7	23.81
1.60	220.3	286.2	24.98

## Data Availability

The original contributions presented in this study are included in the article. Further inquiries can be directed to the corresponding authors.

## Abbreviations

The following abbreviations are used in this manuscript:

AFM	Atomic force microscopy
CCK-8	Cell Counting Kit-8
DMEM	Dulbecco’s Modified Eagle Medium
DSC	Differential scanning calorimetry
FTIR	Fourier transform infrared spectroscopy
*g-g-g*	*gauche-gauche-gauche*
KNPs	Keratin nanoparticles
SEM	Scanning electron microscopy
SFE	Steam flash explosion
TGA	Thermogravimetric analysis
*t-g-t*	*trans-gauche-trans*
